# Cardiac Subsarcolemmal and Interfibrillar Mitochondria Display Distinct Responsiveness to Protection by Diazoxide

**DOI:** 10.1371/journal.pone.0044667

**Published:** 2012-09-04

**Authors:** Ekhson L. Holmuhamedov, Andrew Oberlin, Kevin Short, Andre Terzic, Arshad Jahangir

**Affiliations:** 1 Center for Integrative Research on Cardiovascular Aging, Aurora University of Wisconsin Medical Group, Aurora Health Care, Milwaukee, Wisconsin, United States of American; 2 Division of Cardiovascular Diseases, Mayo Clinic, Rochester, Minnesota, United States of America; 3 Division of Endocrinology, Mayo Clinic, Rochester, Minnesota, United States of America; Universidade Federal do Rio de Janeiro, Brazil

## Abstract

**Objective:**

Cardiac subsarcolemmal (SSM) and interfibrillar (IFM) mitochondrial subpopulations possess distinct biochemical properties and differ with respect to their protein and lipid compositions, capacities for respiration and protein synthesis, and sensitivity to metabolic challenge, yet their responsiveness to mitochondrially active cardioprotective therapeutics has not been characterized. This study assessed the differential responsiveness of the two mitochondrial subpopulations to diazoxide, a cardioprotective agent targeting mitochondria.

**Methods:**

Mitochondrial subpopulations were freshly isolated from rat ventricles and their morphologies assessed by electron microscopy and enzymatic activities determined using standard biochemical protocols with a plate reader. Oxidative phosphorylation was assessed from State 3 respiration using succinate as a substrate. Calcium dynamics and the status of Ca^2+^-dependent mitochondrial permeability transition (MPT) pore and mitochondrial membrane potential were assessed using standard Ca^2+^ and TPP^+^ ion-selective electrodes.

**Results:**

Compared to IFM, isolated SSM exhibited a higher sensitivity to Ca^2+^ overload-mediated inhibition of adenosine triphosphate (ATP) synthesis with decreased ATP production (from 375±25 to 83±15 nmol ATP/min/mg protein in SSM, and from 875±39 to 583±45 nmol ATP/min/mg protein in IFM). In addition, SSM exhibited reduced Ca^2+^-accumulating capacity as compared to IFM (230±13 vs. 450±46 nmol Ca^2+^/mg protein in SSM and IFM, respectively), suggestive of increased Ca^2+^ sensitivity of MPT pore opening. Despite enhanced susceptibility to stress, SSM were more responsive to the protective effect of diazoxide (100 μM) against Ca^2+^ overload-mediated inhibition of ATP synthesis (67% vs. 2% in SSM and IFM, respectively).

**Conclusion:**

These results provide evidence for the distinct sensitivity of cardiac SSM and IFM toward Ca^2+^-dependent metabolic stress and the protective effect of diazoxide on mitochondrial energetics.

## Introduction

Two distinct mitochondrial subpopulations – subsarcolemmal (SSM), situated underneath the sarcolemmal membrane, and interfibrillar (IFM), distributed between myofibrils – have been previously identified in myocardium [1]–[5]. These mitochondrial subpopulations differ in respect to their protein and lipid compositions, capacities for respiration and protein synthesis, and in their sensitivity to metabolic challenge [4]–[13]. Differences in the responsiveness of mitochondrial subpopulations to metabolic stress with enhanced susceptibility of SSM have been demonstrated in the heart [14], [15]. SSM appears to be more vulnerable to ischemic injury and mitochondrial Ca^2+^ overload when compared to IFM [5], [8]–[11], [16]–[20]. Despite distinct biochemical properties and sensitivity to stress, the differences between SSM and IFM in responsiveness to mitochondrially active therapeutics have not been completely characterized. Here, we demonstrate that diazoxide, a cardioprotective mitochondria-targeting agent [21]–[24], effectively protects mitochondria against Ca^2+^ loading and restores Ca^2+^-inhibited oxidative phosphorylation to a greater extent in SSM than in IFM. These results thus provide evidence of distinct sensitivity of cardiac mitochondrial subpopulations toward the protective effect of diazoxide, indicating that SSM could be the preferred target for drug treatment.

## Materials and Methods

### Ethic statement

The study was approved by the Mayo Clinic Institutional Animal Care and use Committee (Protocol # A28201), and all procedures were in accordance with recommendations published in *Guide for the Care and Use of Laboratory Animals*, National Academic Press, Washington, D.C., 1996.

### Mitochondrial isolation

Mitochondria were isolated from the hearts of pentobarbital (100 mg/kg intraperitoneal injection)-anesthetized male adult rats (Sprague-Dawley; Harlan Laboratories, Indianapolis, IN). Following thoracotomy, the heart was rapidly removed from the chest and ventricles were trimmed of atria and connective tissue. The ventricles were placed in ice-cold media containing (in mmol/L): sucrose 50, mannitol 200, KH_2_PO_4_ 5, EGTA 1, 0.2% BSA, MOPS 5 (pH  = 7.3) as described by Holmuhamedov et al. [22]. SSM and IFM were isolated from Polytron®-homogenized (Brinkmann Instruments, Westbury, NY) ventricles using differential centrifugation as previously described [9], [21], [22]. Briefly, isolation of SSM was achieved by mechanical rupture of ventricular tissue with Polytron followed by differential centrifugation, whereas IFM isolation was performed in tissue depleted of SSM by an additional enzymatic digestion with Nagarse and mechanical disruption of residual ventricular tissue to release IFM. A subset of experiments was repeated to rule out nonspecific effect of enzymatic treatment with Nagarse on basic mitochondrial functions in isolated SSM. No significant differences were observed in mitochondrial respiration and Ca^2+^ handling of isolated SSM in the presence or absence of Nagarse treatment. Protein concentration was determined using DC^™^ Protein Determination Kit (Bio-Rad Laboratories, Hercules CA).

### Electron microscopy

SSM and IFM were fixed using Trump's buffer (1% glutaraldehyde, 4% formaldehyde, 0.1-M phosphate buffer, pH 7.2), rinsed and post-fixed in phosphate-buffered 1% osmium tetroxide [25]–[27]. Samples were stained *en bloc* with 2% uranyl acetate for 30 min at 60°C, rinsed, dehydrated, and embedded in Spurr's resin. Thin sections were cut on an Ultracut E ultramicrotome (Reichert-Jung, Vienna, Austria), placed on copper grids and stained with lead citrate. Mitochondria were micrographed with a 1200 EX II electron microscope (Jeol, Tokyo, Japan).

### Citrate synthase activity

The activity of citrate synthase (CS) in SSM and IFM was determined as described by Short et al. [28] with minor modifications. Aliquots of mitochondria were transferred into the incubation buffer, which contained (in mmol/L): 5,5′-dithiobis-(2-nitrobenzoic acid)  = 0.1; acetyl-Co-A  = 0.12; oxaloacetate  = 0.5; TRIZMA  = 100; Triton X-100 = 0.1%; pH  = 8.1. The activity of CS was monitored as absorbance change of 412 nm and expressed as µmoles of thionitrobenzoic acid (TNB)/min/mg protein.

### Western blot

Aliquots of mitochondria solubilized in Laemmle sample buffer were separated on polyacyrlamide gels (Criterion^™^, Bio-Rad Laboratories) and then transferred to polyvinylidene fluoride membranes as described by Short et al. [28]. Briefly, membranes were blocked in 5% milk in Tris-buffered saline with 0.1% Tween-20 for 1 hour and then incubated overnight with primary antibody. Dilutions for the primary antibodies were: citrate synthase 1∶1000 (a kind gift from J.O. Holloszy, MD, Washington University, St. Louis, MO), adenine nucleotide transporter 1 (ANT1) 1∶500 (Santa Cruz Biotechnology, Santa Cruz, CA). Membranes were subsequently exposed to secondary horseradish peroxidase-labeled antibodies at 1∶10,000 (Amersham Biosciences, Piscataway, NJ) and then chemiluminescent substrate (ECL Plus^™^, Amersham) was used for detection. Images captured on Kodak Omat film (Kodak Scientific, Rochester, NY) were then used for densitometry of bands using Kodak Image Station 1000.

### Respiration, membrane potential and calcium transport

Respiration, membrane potential and Ca^2+^ transport of isolated mitochondria were determined using a multichannel system (ABMT-USA, Durham, NC) equipped with oxygen-, tetraphenylphosphonium (TPP^+^)- and Ca^2+^-selective minielectrodes, as previously described [21]–[23]. Briefly, mitochondria (1 mg/ml) were added into the incubation buffer containing (in mM): KCl 110, K_2_HPO_4_ 5, succinate 5, pyruvate 5, and MOPS 10 (pH  = 7.35) and respiration was measured using calibrated Clark-type O2 minielectrode. Mitochondrial membrane potential was measured simultaneously with respiration using TPP^+^-sensitive minielectrode, manufactured and calibrated as described by Kamo et al. [29]. Concentration of TPP^+^ was 200 nM, and mitochondrial membrane potential was calculated as previously described [21], [22], [29]. Mitochondrial Ca^2+^ uptake was measured from changes in the free Ca^2+^ concentration within the suspension using calibrated Ca^2+^-selective minielectrodes (Microelectrodes Inc., Bedford, NH) as described [22], [25]. Mitochondrial Ca^2+^-accumulating capacity was determined as the total amount of Ca^2+^ accumulated into the matrix from a train of 50-μM Ca^2+^ pulses added at 1-min intervals until the load reached a threshold pulse after which mitochondria underwent irreversible and rapid Ca^2+^ release [30], [31].

### Adenosine triphosphate synthesis

Adenosine triphosphate (ATP) production in mitochondria was determined using K_2_CO_3_/MOPS-neutralized HClO_4_-soluble mitochondrial extracts by high-pressure liquid chromatography (Hewlett-Packard, Waldbronn, Germany) as described by Holmuhamedov et al. [23]. Briefly, 200 μl of mitochondrial suspension were treated with 20 μl of 3.3-M HClO_4_, and precipitated proteins were removed by centrifugation (60 s, 14,000 rpm, 4°C). After neutralization of the supernatant with 80 μl of a mixture containing 2.5-M K_2_CO_3_ in 1-M HEPES, the precipitate was separated by centrifugation (60 s, 14,000 rpm, 4°C), and the concentration of ATP within the extract was determined in coupled enzymatic reactions [32]. The time course of adenosine diphosphate (ADP)-to-ATP conversion within mitochondrial suspension was monitored from changes in NADPH fluorescence (Ascent FL, Scientific Resources, Saint Paul, MN) in a coupled hexokinase/glucose-6-phosphate dehydrogenase assay [21]–[23], [32].

### Drugs

Diazoxide (Research Biochemical International, Natick, MA) was dissolved as a concentrated stock solution in dimethylsulfoxide (DMSO), and the maximal concentration of DMSO in the incubation medium was kept under 0.5%. All other chemicals were from Sigma Chemicals (St. Louis, MO).

### Statistical analysis

Data are expressed as mean ± standard error of mean, and “n” represents the number of mitochondrial isolations. Comparison between groups was made using analysis of variance (ANOVA) with post-hoc test. ANOVA was performed for multiple comparisons between groups using two-way comparison of means by Tukey-Kramer HSD test, and p<0.05 was considered to be statistically significant.

## Results

### Biochemical similarity and differences in isolated cardiac SSM and IFM

**Figure 1:**
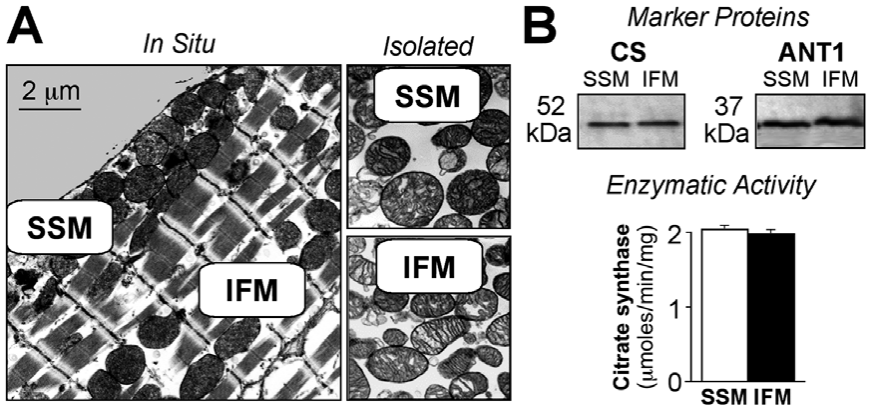
Morphological and biochemical characteristics of two mitochondrial populations in heart muscle. A, Electron micrographs of subsarcolemmal (SSM) and interfibrillar (IFM) mitochondria within the heart cell (*In Situ*) and after isolation (*Isolated*). Magnification: x10, 000. B, Expression of mitochondria-specific marker proteins in SSM and IFM. Top panel displays Western blots of citrate synthase (CS) and adenine nucleotide transporter 1 (ANT1). Bottom panel displays bar graphs of the activity of CS (SSM, *open bars*; IFM, *filled bars*).

Electron micrographs of the heart muscle demonstrate intracellular localization of mitochondrial subpopulations and morphological appearance of SSM and IFM *in situ* and after isolation (Fig. 1A). Intracellular SSM have a round shape and a less electron-dense “light” matrix, while IFM are elongated and rod-shaped with the matrix containing a greater electron-dense material (Fig. 1A). The content of CS and ANT1, specific mitochondrial matrix and membrane proteins were all determined by Western blot (Fig. 1B, *top panels*). Both subpopulations of isolated mitochondria demonstrated similar levels of expression of CS and ANT1 (Fig. 1B, *top panels*). In addition, the activity of CS in mitochondrial subpopulations was similar (2.04±0.03 vs. 1.98±0.04 µmoles TNB/min/mg protein in SSM and IFM, respectively; Fig. 1B, *lower panel*, n = 6, p = NS).

### Ca^2+^ handling and oxidative phosphorylation capacity of SSM and IFM

**Figure 2:**
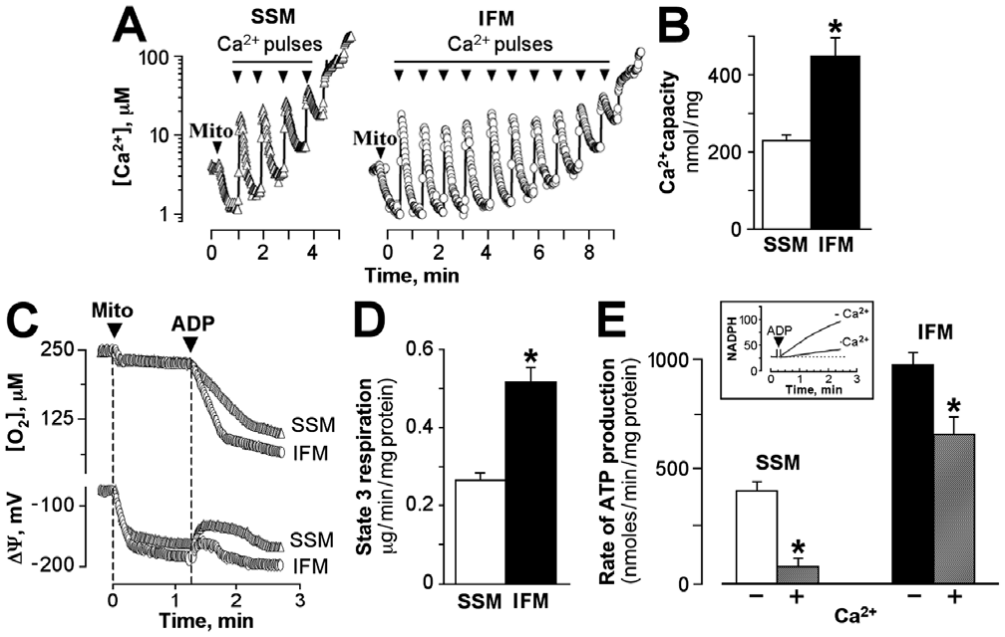
Ca^2+^ handling and oxidative phosphorylation capacity of subsarcolemmal (SSM) and interfibrillar (IFM) mitochondria. A, Typical tracing of Ca^2+^ loading in SSM (*left panel*) and IFM (*right panel*). Mitochondria were loaded with consecutive Ca^2+^ pulses (*arrows*), and each pulse delivers 50 nmol of Ca^2+^. B, Average Ca^2+^-accumulating capacity of SSM (*open bar*) and IFM (*filled bar*), n = 6, p<0.05. C, Typical tracing of ADP-induced changes in oxygen consumption (*top*) and changes in membrane potential from baseline (*bottom*) of SSM and IFM. D, Average ADP-stimulated (State 3) respiration of SSM and IFM (n = 6, p<0.05). E, The rate of ATP production in SSM (*left bars*) and IFM (*right bars*) before (*B/W bars*) and after (*gray bars*) loading with 150 nmol Ca^2+^/mg protein, n = 3, p<0.05. *Inset*: Alternative monitoring of ATP production in mitochondria using coupled enzymes. Asterisks signify statistical differences with p<0.05.

The sensitivity of mitochondria toward Ca^2+^-induced mitochondrial permeability transition (MPT) pore opening was determined from the number of Ca^2+^ pulses required to reach the threshold for rapid and spontaneous Ca^2+^ release [31]. There was no difference in the baseline content of endogenous Ca^2+^ in isolated SSM and IFM measured immediately after isolation (2.1±0.9 vs. 2.2±1.1 nmol Ca^2+^/mg protein, respectively, n = 6, data not shown). However, the maximal Ca^2+^-accumulating capacity of mitochondrial subpopulations (determined from experiments with multiple Ca^2+^ pulses, described in Materials and Methods) was significantly decreased in SSM and was 230±13 nmol Ca^2+^/mg protein as compared with 450±46 nmol Ca^2+^/mg protein in IFM (Fig. 2A and 2B, n = 6, p<0.05).

The capacity for oxidative phosphorylation assessed from ADP-mediated increase in mitochondrial respiration and membrane depolarization was also different in SSM and IFM (Fig. 2C and 2D). On average, ADP-stimulated respiration (State 3) in SSM was 49% lower than in IFM (267±15 vs. 518±37 ng-atoms O_2_/min/mg protein, respectively; Fig. 2D, n = 6, p<0.05). Decreased State 3 respiration in SSM correlated with a longer period of ADP-to-ATP conversion, as monitored from reversible and transient ADP-induced membrane depolarization from the baseline (76±3 sec in SSM versus 36±3 sec in IFM; Fig. 2C, *lower panel*). Although the rates of respiration upon completion of ADP-to-ATP conversion (State 4) were different in these populations (67±5 and 123±11 ng-atoms O_2_/min/mg protein in SSM and IFM, respectively), the resulting respiratory control ratio was not significantly different (3.2±0.7 vs. 4.2±0.5, n = 6), demonstrating similar coupling of oxidative phosphorylation in SSM and IFM.

Loading of mitochondria with 150 nmol Ca^2+^/mg protein inhibited oxidative phosphorylation in both SSM and IFM, but the extent of inhibition was significantly higher in SSM. The rate of ATP synthesis in the presence of Ca^2+^ was decreased from 375±25 to 83±15 nmol ATP/min/mg protein in SSM, and from 875±39 to 583±45 nmol ATP/min/mg protein in IFM (Fig. 2E, n = 6, p<0.05). Thus, compared to IFM, SSM demonstrated lesser Ca^2+^ handling capacity, enhanced susceptibility to MPT pore opening, decreased rate of oxidative phosphorylation and enhanced sensitivity to inhibition of mitochondrial energetics by excessive Ca^2+^ loading.

### Diazoxide decreases Ca^2+^ loading preferentially in SSM

**Figure 3:**
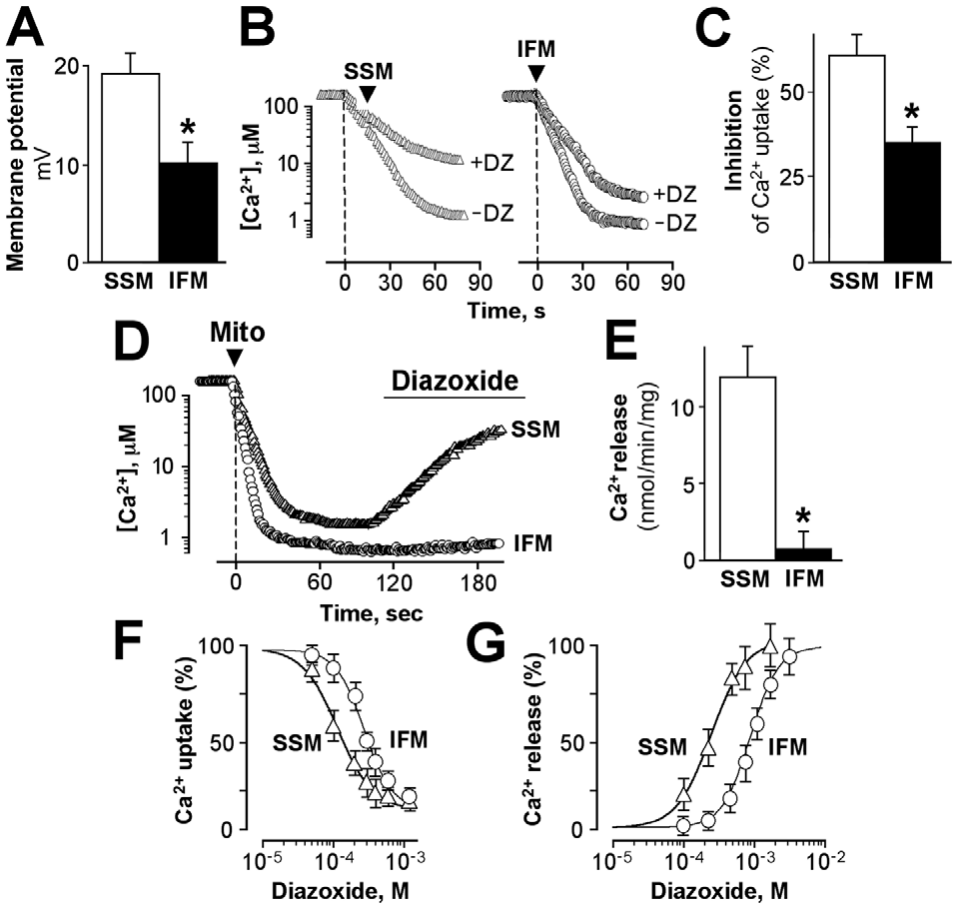
Effect of diazoxide on the membrane potential and Ca^2+^ handling in subsarcolemmal (SSM) and interfibrillar (IFM) mitochondria. A, Depolarizing effect of diazoxide (100 µM) in SSM (*open bar*) and IFM (*filled bar*), n = 6, p<0.05. B, Mitochondrial Ca^2+^ uptake in SSM and IFM in the absence (-DZ) and presence (+DZ) of diazoxide (100 µM). C, Inhibition of the rate of Ca^2+^ uptake in SSM (*open bar*) and IFM (*filled bar*), n = 6, p<0.05. D, Diazoxide (100 µM) mediated Ca^2+^ release from preloaded SSM and IFM. E, Average rate of diazoxide-mediated Ca^2+^ release from mitochondria, n = 3, p<0.05. F and G, Dose-dependent effect of diazoxide on Ca^2+^ uptake (*F*) and Ca^2+^ release (*G*) in SSM (*triangles*) and IFM (*circles*), n = 6.

Diazoxide, an opener of sarcolemmal ATP-sensitive K^+^ channels with cardioprotective properties also known to target and depolarize isolated mitochondria [21], [22], [33]–[36], differentially affected mitochondrial membrane potential and Ca^2+^ handling in these two mitochondrial subpopulations. The SSM-oxidizing succinate, demonstrated a higher sensitivity to diazoxide (100 μM)-induced depolarization of the inner membrane (19±2 mV) compared with IFM (10±2 mV; Fig. 3A, n = 6, p<0.05). Similarly, when added to mitochondria prior to Ca^2+^ loading, diazoxide (100 µM) suppressed the rate of Ca^2+^ uptake preferentially in SSM (from 347±9 to 137±7 nmol Ca^2+^/min/mg protein) than in IFM (from 503±13 to 326±10 nmol Ca^2+^/min/mg protein), demonstrating a 61% vs. 35% inhibition of Ca^2+^ uptake in SSM and IFM, respectively (Fig. 3B and 3C, n = 6, p<0.05). In the absence of diazoxide, neither SSM nor IFM demonstrated release of accumulated Ca^2+^ during 20 min of observation (data not shown). However, diazoxide had a differential effect on Ca^2+^ release from Ca^2+^-loaded mitochondria. In mitochondria preloaded with 100 nmol Ca^2+^/mg protein, the same concentration of diazoxide (100 µM) induced faster release of accumulated Ca^2+^ from SSM than from IFM (Fig. 3D). On average, the rate of diazoxide-induced Ca^2+^ release was fivefold higher (10±2 vs. 2±1 nmol Ca^2+^/min/mg protein) in SSM and IFM, respectively (Fig. 3E, n = 6, p<0.05). Overall, the effect of diazoxide on mitochondrial Ca^2+^ uptake and release was dose-dependent, and the concentration of diazoxide causing 50% inhibition of Ca^2+^ uptake (IC_50_) was estimated at 104±15 and 289±32 µM in SSM and IFM, respectively (Fig. 3F, n = 6, p<0.05), whereas the concentration of diazoxide causing 50% Ca^2+^ release was 114±21 and 377±44 µM in SSM and IFM, respectively (Fig. 3G, n = 6, p<0.05). Thus, SSM and IFM exhibit differential responsiveness toward diazoxide-mediated membrane depolarization, Ca^2+^ uptake, and release of accumulated Ca^2+^.

### Diazoxide restores Ca^2+^-inhibited ATP production

**Figure 4:**
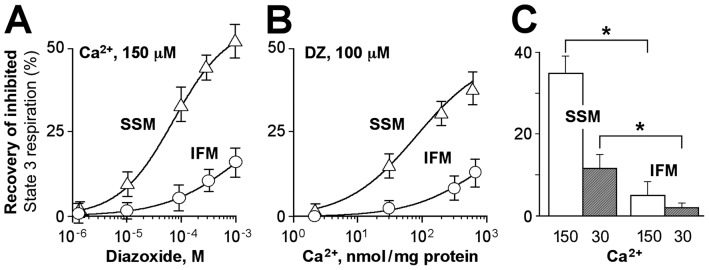
Diazoxide-mediated recovery of Ca^2+^-inhibited ATP synthesis. A, Dose-dependent effect of diazoxide on restoration of Ca^2+^-inhibited State 3 respiration in subsarcolemmal (SSM) (*triangles*) and interfibrillar (IFM) (*circles*) mitochondria. B, Effect of diazoxide on Ca^2+^-inhibited State 3 respiration in SSM and IFM preloaded with (0–150 nmol Ca^2+^/mg protein). C, Bar graphs of diazoxide (100 µM)-mediated recovery of Ca^2+^-inhibited State 3 respiration in SSM and IFM loaded with 150 (*open bars*) and 30 nmol Ca^2+^/mg protein (*hatched bars*), n = 3. Asterisks signify statistical differences with p<0.05.

Excessive mitochondrial Ca^2+^ loading inhibited oxidative phosphorylation (Fig. 2) and diazoxide in a dose-dependent manner restored State 3 respiration in both SSM and IFM (Fig. 4A). The rescuing effect of diazoxide on Ca^2+^-inhibited State 3 respiration was more prominent in SSM compared to IFM (Fig. 4) and was dependent upon the level of mitochondrial Ca^2+^ overload (Fig. 4B and 4C). In mitochondria loaded with 150 nmol Ca^2+^/mg protein, diazoxide restored Ca^2+^-inhibited ADP-stimulated respiration by 35±4% in SSM compared with 5±3% in IFM (Fig. 4C, p<0.05, n = 6, *open bars*), whereas at 30 nmol Ca^2+^/mg protein, the protective effect of diazoxide was only by 12±3% and 4±0.9% in SSM and IFM, respectively (Fig. 4C, n = 6, *hatched bars*). ATP synthesis in mitochondria, monitored using coupled enzymatic reactions [22], [23], confirmed that diazoxide was more efficient in recovering the rate of Ca^2+^-inhibited ATP production in SSM than IFM. In mitochondria loaded with 150 nmol Ca^2+^/mg protein, diazoxide restored the rate of ATP production by 48±5% in SSM compared with 5±3% in IFM. Thus, diazoxide restores Ca^2+^-inhibited mitochondrial ATP synthesis in both SSM and IFM populations, but the magnitude of this protective effect is markedly higher in SSM compared to IFM.

## Discussion

This study demonstrates that SSM and IFM, two subpopulations of cardiac mitochondria, exhibit differential susceptibility to Ca^2+^-dependent inhibition of oxidative phosphorylation, opening of MPT pore and sensitivity to the protective effect of diazoxide, a mitochondrially active cardioprotective agent. These results provide additional insights into the functional and pharmacological differences between the mitochondrial subpopulations previously shown to differ in their biochemical characteristics, protein and lipid composition, and susceptibility toward metabolic challenge [6], [8]–[12], [17], [37]. Here we demonstrate that SSM were more vulnerable to the damaging effects of Ca^2+^ overload and inhibition of oxidative phosphorylation when compared to IFM, in line with previous observations [7], [10], [11], [16]–[18]. While Ca^2+^-mediated inhibition of oxidative phosphorylation was more prominent in SSM compared to IFM [8], [10], diazoxide was more effective in restoring Ca^2+^-inhibited oxidative phosphorylation in SSM than IFM (Figs. 3 and 4). This finding could be of great clinical relevance, as SSM has been shown to be more susceptible to injury than the IFM [6], [10], [11], [17], [38]. Mitochondrial energy production is determined by the activity of key enzymes of tricarboxylic cycle, which are regulated by Ca^2+^ ions in the physiological range of Ca^2+^ concentrations [39], [40]. However, excessive Ca^2+^ loading under pathological conditions has a detrimental effect on mitochondrial ATP synthesis [39]–[44]. Diazoxide has been demonstrated to protect mitochondrial energetic function and preserve cellular ATP level under metabolic stress [22], [45]–[48]. Here, we demonstrate that diazoxide-mediated decrease in mitochondrial Ca^2+^ loading is accompanied by partial restoration of Ca^2+^-inhibited ATP production in both mitochondrial subsets, but the responsiveness of SSM to diazoxide was much greater. The levels of expression of the mitochondrial-specific matrix enzyme (citrate synthase) and membrane protein (adenine nucleotide transporter) were not different in the two subpopulations, suggesting that distinct properties were not introduced due to differences in the isolation protocol or the number of mitochondria but are intrinsic features of mitochondrial subpopulations. The salvaging effect of diazoxide on ATP production in Ca^2+^-loaded mitochondria was greater in mitochondria with a higher level of Ca^2+^ load, indicating that the effect of diazoxide is condition-selective, and it is rather the release of inhibited oxidative phosphorylation than the activation of mitochondrial ATP synthesis. In accordance with this notion is the fact that in the absence of Ca^2+^ loading, diazoxide had little effect on State 3 respiration in both populations, and may even slow the rate of ATP production as reported previously [22], [23], [33], [49], [50].

The precise mechanism of diazoxide action on mitochondria *in vivo* remains unknown, [26], [33], [34], [46], [47], [51] and likely involves multiple effects, including mitochondrial uncoupling by a protonophoric effect [24], potassium transport, and substrate metabolism reported in isolated rat hearts [24], [33], [34], [36], [50], [52] and mitochondria [23], [26]. Additional factors may influence the overall effect of diazoxide on cardiac energetics and protection, including the intracellular locale of biochemically and functionally different mitochondrial subpopulations exposed to a different degree of Ca^2+^ load and metabolic stress. This is of significance in view of the different responsiveness of subsarcolemmal and interfibrillar mitochondria in intact cardiomyocytes [8]–[11], [16], [17], [53] and the heterogeneity in mitochondrial Ca^2+^ loading demonstrated in various cellular microdomains [54]–[58]. Our observation that SSM are more sensitive toward diazoxide-mediated protection from Ca^2+^ injury is consistent with reports on the higher vulnerability and reduced tolerance of this mitochondrial population toward Ca^2+^-mediated functional and structural damages. Therefore, our observation indicates that by preferentially targeting SSM (the more vulnerable subpopulation of cardiac mitochondria) diazoxide will be protective against ischemia/reperfusion-mediated injury.

**Figure 5:**
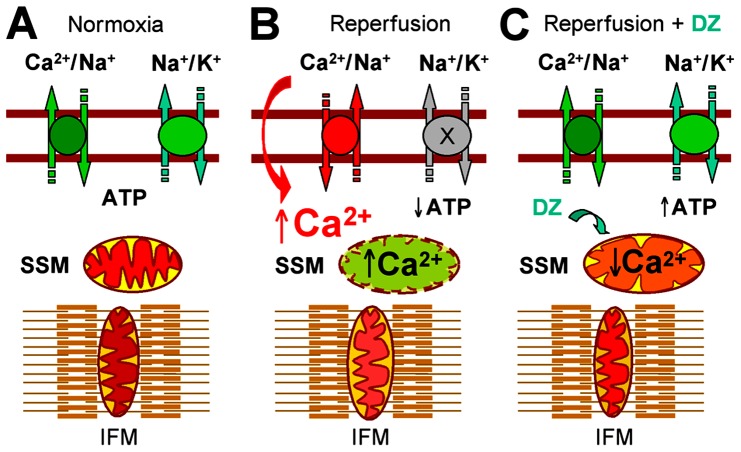
Schematic illustration of preferential targeting of subsarcolemmal mitochondria (SSM) by diazoxide. A, Before ischemic insult (*Normoxia*), SSM and interfibrillar mitochondria (IFM) produce sufficient ATP to feed ionic pumps and maintain ionic homeostasis of cell. During ischemic period and decreased supply of substrates and oxygen, the mitochondrial ATP formation decreases and disables ionic pumps, resulting in increased cytosolic Ca^2+^. B, At reperfusion (*Reperfusion*), restored supply of substrates and oxygen reenergizes the SSM, resulting in excessive Ca^2+^ uptake (from high Ca^2+^ cytosol) and inhibition of ATP production. C, In the presence of diazoxide during reperfusion (*Reperfusion + DZ*) Ca^2+^ uptake into SSM will be decreased, thus preserving mitochondrial ability to produce ATP, which is required for the activity of ionic pumps and restoration of normal cellular homeostasis.

Under normal conditions, both SSM and IFM are efficient in meeting demands of the cellular ATP-dependent processes and maintaining ionic homeostasis of cells (Fig. 5A). During ischemic insult and decreased delivery of oxygen, mitochondrial ATP production drops and ionic pumps fail to maintain required gradients of Na^+^ and K^+^ ions across the sarcolemmal membrane, resulting in increased cytosolic Ca^2+^ (Fig. 5B). At reperfusion, oxygen availability quickly restores mitochondrial membrane potential and leads to excessive uptake of Ca^2+^ from the cytosol. By promoting MPT pore opening or inhibition of oxidative phosphorylation, this oxygen availability causes greater injury to the more vulnerable SSM than IFM, resulting in additional structural and functional derangements that limit the capacity of SSM, which are located in the close vicinity of plasma membrane ionic pumps [4], [5], [9]–[11], [13] in order to synthesize ATP, a critical function for maintaining homeostasis at the time of reperfusion (Fig. 5B). From our findings, we speculate that during and/or following metabolic stress, cardioprotective diazoxide moderately depolarizes mitochondria and prevents SSM against excessive Ca^2+^ overload by decreasing the rate of Ca^2+^ uptake or releasing accumulated Ca^2+^ or both, resulting in preservation of ATP production in the more vulnerable and strategically distributed SSM (Fig. 5), thus rescuing the energy source for ATP-dependent cellular processes, such as the maintenance of transsarcolemmal ionic homeostasis.

In summary, SSM are a preferential target for the cardioprotective drug diazoxide in the setting of ischemia and heart reperfusion. Our data suggest that the mechanism of protective action of diazoxide could be through decreased Ca^2+^ uptake, reduction of mitochondrial Ca^2+^ loading and through release of excessively loaded Ca^2+^ and restoration of Ca^2+^-inhibited ATP production in postischemic heart muscle.

## References

[1] Palmer JW, Tandler B, Hoppel CL (1977) Biochemical properties of subsarcolemmal and interfibrillar mitochondria isolated from rat cardiac muscle. J Biol Chem 252: 8731–8739. 925018

[2] Duan J, Karmazyn M (1989) Relationship between oxidative phosphorylation and adenine nucleotide translocase activity of two populations of cardiac mitochondria and mechanical recovery of ischemic hearts following reperfusion. Can J Physiol Pharmacol 67: 704–709. 2548694 10.1139/y89-114

[3] Hoppel CL, Moghaddas S, Lesnefsky EJ (2002) Interfibrillar cardiac mitochondrial comples III defects in the aging rat heart. Biogerontology 3: 41–44. 12014840 10.1023/a:1015251212039

[4] Hoppel CL, Tandler B, Fujioka H, Riva A (2009) Dynamic organization of mitochondria in human heart and in myocardial disease. Int J Biochem Cell Biol 41: 1949–1956. 19446651 10.1016/j.biocel.2009.05.004 PMC3221317

[5] Riva A, Tandler B, Loffredo F, Vazquez E, Hoppel C (2005) Structural differences in two biochemically defined populations of cardiac mitochondria. Am J Physiol Heart Circ Physiol 289: H868–H872. 15821034 10.1152/ajpheart.00866.2004

[6] Duan JM, Karmazyn M (1989) Acute effects of hypoxia and phosphate on two populations of heart mitochondria. Mol Cell Biochem 90: 47–56. 2608032 10.1007/BF00225220

[7] Lesnefsky EJ, Slabe TJ, Stoll MS, Minkler PE, Hoppel CL (2001) Myocardial ischemia selectively depletes cardiolipin in rabbit heart subsarcolemmal mitochondria. Am J Physiol Heart Circ Physiol 280: H2770–H2778. 11356635 10.1152/ajpheart.2001.280.6.H2770

[8] McMillin-Wood J, Wolkowicz PE, Chu A, Tate CA, Goldstein MA, et al (1980) Calcium uptake by two preparations of mitochondria from heart. Biochim Biophys Acta 591: 251–265. 7397124 10.1016/0005-2728(80)90157-7

[9] Palmer JW, Tandler B, Hoppel CL (1985) Biochemical differences between subsarcolemmal and interfibrillar mitochondria from rat cardiac muscle: effects of procedural manipulations. Arch Biochem Biophys 236: 691–702. 2982322 10.1016/0003-9861(85)90675-7

[10] Palmer JW, Tandler B, Hoppel CL (1986) Heterogeneous response of subsarcolemmal heart mitochondria to calcium. Am J Physiol 250: H741–H748. 3706549 10.1152/ajpheart.1986.250.5.H741

[11] Piper HM, Sezer O, Schleyer M, Schwartz P, Hütter JF, et al (1985) Development of ischemia-induced damage in defined mitochondrial subpopulations. J Mol Cell Cardiol 17: 885–896. 4046049 10.1016/s0022-2828(85)80102-4

[12] Servais S, Couturier K, Koubi H, Rouanet JL, Desplanches D, et al (2003) Effect of voluntary exercise on H2O2 release by subsarcolemmal and intermyofibrillar mitochondria. Free Radic Biol Med 35: 24–32. 12826253 10.1016/s0891-5849(03)00177-1

[13] Williamson CL, Dabkowski ER, Baseler WA, Croston TL, Alway SE, et al (2010) Enhanced apoptotic propensity in diabetic cardiac mitochondria: influence of subcellular spatial location. Am J Physiol Heart Circ Physiol 298: H633–H642. 19966057 10.1152/ajpheart.00668.2009 PMC2822591

[14] Kavazis AN, McClung JM, Hood DA, Powers SK (2008) Exercise induces a cardiac mitochondrial phenotype that resists apoptotic stimuli. Am J Physiol Heart Circ Physiol 294: H928–H935. 18083894 10.1152/ajpheart.01231.2007

[15] Kavazis AN, Alvarez S, Talbert E, Lee Y, Powers SK (2009) Exercise training induces a cardioprotective phenotype and alterations in cardiac subsarcolemmal and intermyofibrillar mitochondrial proteins. Am J Physiol Heart Circ Physiol 297: H144–H152. 19429812 10.1152/ajpheart.01278.2008 PMC2711746

[16] Lesnefsky EJ, Moghaddas S, Tandler B, Kerner J, Hoppel CL (2001) Mitochondrial dysfunction in cardiac disease: ischemia – reperfusion, aging, and heart failure. J Mol Cell Cardiol 33: 1065–1089. 11444914 10.1006/jmcc.2001.1378

[17] Lesnefsky EJ, Tandler B, Ye J, Slabe TJ, Turkaly J, et al (1997) Myocardial ischemia decreases oxidative phosphorylation through cytochrome oxidase in subsarcolemmal mitochondria. Am J Physiol 273: H1544–H1554. 9321848 10.1152/ajpheart.1997.273.3.H1544

[18] Judge S, Leeuwenburgh C (2007) Cardiac mitochondrial bioenergetics, oxidative stress, and aging. Am J Physiol Cell Physiol 292: C1983–C1992. 17344313 10.1152/ajpcell.00285.2006

[19] Judge S, Jang YM, Smith A, Hagen T, Leeuwenburgh C (2005) Age-associated increases in oxidative stress and antioxidant enzyme activities in cardiac interfibrillar mitochondria: implications for the mitochondrial theory of aging. FASEB J 19: 419–421. 15642720 10.1096/fj.04-2622fje

[20] Bizeau ME, Willis WT, Hazel JR (1998) Differential responses to endurance training in subsarcolemmal and intermyofibrillar mitochondria. J Appl Physiol 85: 1279–1284. 9760317 10.1152/jappl.1998.85.4.1279

[21] Holmuhamedov EL, Jovanović S, Dzeja PP, Jovanović A, Terzic A (1998) Mitochondrial ATP-sensitive K+ channels modulate cardiac mitochondrial function. Am J Physiol 275: H1567–H1576. 9815062 10.1152/ajpheart.1998.275.5.H1567

[22] Holmuhamedov EL, Wang L, Terzic A (1999) ATP-sensitive K+ channel openers prevent Ca2+ overload in rat cardiac mitochondria. J Physiol 519 Pt 2: 347–360. 10.1111/j.1469-7793.1999.0347m.x PMC2269505 10457054

[23] Holmuhamedov EL, Ozcan C, Jahangir A, Terzic A (2001) Restoration of Ca2+-inhibited oxidative phosphorylation in cardiac mitochondria by mitochondrial Ca2+ unloading. Mol Cell Biochem 220: 135–140. 11451373 10.1023/a:1010894427373

[24] Holmuhamedov EL, Jahangir A, Oberlin A, Komarov A, Colombini M, et al (2004) Potassium channel openers are uncoupling protonophores: implication in cardioprotection. FEBS Lett 568: 167–170. 15196941 10.1016/j.febslet.2004.05.031

[25] Jahangir A, Ozcan C, Holmuhamedov EL, Terzic A (2001) Increased calcium vulnerability of senescent cardiac mitochondria: protective role for a mitochondrial potassium channel opener. Mech Ageing Dev 122: 1073–1086. 11389925 10.1016/s0047-6374(01)00242-1

[26] Ozcan C, Holmuhamedov EL, Jahangir A, Terzic A (2001) Diazoxide protects mitochondria from anoxic injury: implications for myopreservation. J Thorac Cardiovasc Surg 121: 298–306. 11174735 10.1067/mtc.2001.111421

[27] Preston CC, Oberlin AS, Holmuhamedov EL, Gupta A, Sagar S, et al (2008) Aging-induced alterations in gene transcripts and functional activity of mitochondrial oxidative phosphorylation complexes in the heart. Mech Ageing Dev 129: 304–312. 18400259 10.1016/j.mad.2008.02.010 PMC2464774

[28] Short KR, Bigelow ML, Kahl J, Singh R, Coenen-Schimke J, et al (2005) Decline in skeletal muscle mitochondrial function with aging in humans. Proc Natl Acad Sci U S A 102: 5618–5623. 15800038 10.1073/pnas.0501559102 PMC556267

[29] Kamo N, Muratsugu M, Hongoh R, Kobatake Y (1979) Membrane potential of mitochondria measured with an electrode sensitive to tetraphenyl phosphonium and relationship between proton electrochemical potential and phosphorylation potential in steady state. J Membr Biol 49: 105–121. 490631 10.1007/BF01868720

[30] Bernardi P, Krauskopf A, Basso E, Petronilli V, Blachly-Dyson E, et al (2006) The mitochondrial permeability transition from in vitro artifact to disease target. FEBS J 273: 2077–2099. 16649987 10.1111/j.1742-4658.2006.05213.x

[31] Argaud L, Gateau-Roesch O, Chalabreysse L, Gomez L, Loufouat J, et al (2004) Preconditioning delays Ca2+-induced mitochondrial permeability transition. Cardiovasc Res 61: 115–122. 14732208 10.1016/j.cardiores.2003.11.003

[32] He ZH, Chillingworth RK, Brune M, Corrie JE, Trentham DR, et al (1997) ATPase kinetics on activation of rabbit and frog permeabilized isometric muscle fibres: a real time phosphate assay. J Physiol 501 (Pt 1): 125–148. 10.1111/j.1469-7793.1997.125bo.x PMC1159509 9174999

[33] Grover GJ, Garlid KD (2000) ATP-Sensitive potassium channels: a review of their cardioprotective pharmacology. J Mol Cell Cardiol 32: 677–695. 10756123 10.1006/jmcc.2000.1111

[34] Garlid KD, Paucek P, Yarov-Yarovoy V, Murray HN, Darbenzio RB, et al (1997) Cardioprotective effect of diazoxide and its interaction with mitochondrial ATP-sensitive K+ channels. Possible mechanism of cardioprotection. Circ Res 81: 1072–1082. 9400389 10.1161/01.res.81.6.1072

[35] Costa AD, Garlid KD (2009) MitoKATP activity in healthy and ischemic hearts. J Bioenerg Biomembr 41: 123–126. 19353252 10.1007/s10863-009-9213-y

[36] Gross GJ, Fryer RM (1999) Sarcolemmal versus mitochondrial ATP-sensitive K+ channels and myocardial preconditioning. Circ Res 84: 973–979. 10325234 10.1161/01.res.84.9.973

[37] Suh JH, Heath SH, Hagen TM (2003) Two subpopulations of mitochondria in the aging rat heart display heterogenous levels of oxidative stress. Free Radic Biol Med 35: 1064–1072. 14572609 10.1016/s0891-5849(03)00468-4 PMC4696537

[38] Ruiz-Meana M, Garcia-Dorado D, Miró-Casas E, Abellán A, Soler-Soler J (2006) Mitochondrial Ca2+ uptake during simulated ischemia does not affect permeability transition pore opening upon simulated reperfusion. Cardiovasc Res 71: 715–724. 16860295 10.1016/j.cardiores.2006.06.019

[39] McCormack JG, Denton RM (1989) The role of Ca2+ ions in the regulation of intramitochondrial metabolism and energy production in rat heart. Mol Cell Biochem 89: 121–125. 2682206

[40] McCormack JG, Denton RM (1993) The role of intramitochondrial Ca2+ in the regulation of oxidative phosphorylation in mammalian tissues. Biochem Soc Trans 21 (Pt 3): 793–799. 10.1042/bst0210793 8224512

[41] De Gómez-Puyou MT, Gavilanes M, Gómez-Puyou A, Ernster L (1980) Control of activity states of heart mitochondrial ATPase. Role of the proton-motive force and Ca2+. Biochim Biophys Acta 592: 396–405. 6448069 10.1016/0005-2728(80)90087-0

[42] Ferrari R, Pedersini P, Bongrazio M, Gaia G, Bernocchi P, et al (1993) Mitochondrial energy production and cation control in myocardial ischaemia and reperfusion. Basic Res Cardiol 88: 495–512. 8117254 10.1007/BF00795415

[43] Miyata H, Lakatta EG, Stern MD, Silverman HS (1992) Relation of mitochondrial and cytosolic free calcium to cardiac myocyte recovery after exposure to anoxia. Circ Res 71: 605–613. 1499108 10.1161/01.res.71.3.605

[44] Griffiths EJ, Rutter GA (2009) Mitochondrial calcium as a key regulator of mitochondrial ATP production in mammalian cells. Biochim Biophys Acta 1787: 1324–1333. 19366607 10.1016/j.bbabio.2009.01.019

[45] Sato T, Marbán E (2000) The role of mitochondrial K(ATP) channels in cardioprotection. Basic Res Cardiol 95: 285–289. 11005583 10.1007/s003950070047

[46] Kowaltowski AJ, Seetharaman S, Paucek P, Garlid KD (2001) Bioenergetic consequences of opening the ATP-sensitive K(+) channel of heart mitochondria. Am J Physiol Heart Circ Physiol 280: H649–H657. 11158963 10.1152/ajpheart.2001.280.2.H649

[47] Dos Santos P, Kowaltowski AJ, Laclau MN, Seetharaman S, Paucek P, et al (2002) Mechanisms by which opening the mitochondrial ATP-sensitive K(+) channel protects the ischemic heart. Am J Physiol Heart Circ Physiol 283: H284–H295. 12063301 10.1152/ajpheart.00034.2002

[48] Akao M, O'Rourke B, Kusuoka H, Teshima Y, Jones SP, et al (2003) Differential actions of cardioprotective agents on the mitochondrial death pathway. Circ Res 92: 195–202. 12574147 10.1161/01.res.0000051862.16691.f9

[49] Tanonaka K, Taguchi T, Koshimizu M, Ando T, Morinaka T, et al (1999) Role of an ATP-sensitive potassium channel opener, YM934, in mitochondrial energy production in ischemic/reperfused heart. J Pharmacol Exp Ther 291: 710–716. 10525091

[50] Fryer RM, Eells JT, Hsu AK, Henry MM, Gross GJ (2000) Ischemic preconditioning in rats: role of mitochondrial K(ATP) channel in preservation of mitochondrial function. Am J Physiol Heart Circ Physiol 278: H305–H312. 10644614 10.1152/ajpheart.2000.278.1.H305

[51] Garlid KD, Costa AD, Quinlan CL, Pierre SV, Dos Santos P (2009) Cardioprotective signaling to mitochondria. J Mol Cell Cardiol 46: 858–866. 19118560 10.1016/j.yjmcc.2008.11.019 PMC2683183

[52] Minners J, Lacerda L, Yellon DM, Opie LH, McLeod CJ, et al (2007) Diazoxide-induced respiratory inhibition – a putative mitochondrial K(ATP) channel independent mechanism of pharmacological preconditioning. Mol Cell Biochem 294: 11–18. 17136444 10.1007/s11010-005-9066-6

[53] Gallitelli MF, Schultz M, Isenberg G, Rudolf F (1999) Twitch-potentiation increases calcium in peripheral more than in central mitochondria of guinea-pig ventricular myocytes. J Physiol 518 (Pt 2): 433–447. 10.1111/j.1469-7793.1999.0433p.x PMC2269425 10381590

[54] Rizzuto R, Brini M, Murgia M, Pozzan T (1993) Microdomains with high Ca2+ close to IP3-sensitive channels that are sensed by neighboring mitochondria. Science 262: 744–747. 8235595 10.1126/science.8235595

[55] Rizzuto R, Bastianutto C, Brini M, Murgia M, Pozzan T (1994) Mitochondrial Ca2+ homeostasis in intact cells. J Cell Biol 126: 1183–1194. 8063855 10.1083/jcb.126.5.1183 PMC2120160

[56] Rutter GA, Burnett P, Rizzuto R, Brini M, Murgia M, et al (1996) Subcellular imaging of intramitochondrial Ca2+ with recombinant targeted aequorin: significance for the regulation of pyruvate dehydrogenase activity. Proc Natl Acad Sci U S A 93: 5489–5494. 8643602 10.1073/pnas.93.11.5489 PMC39273

[57] Montero M, Alonso MT, Albillos A, Cuchillo-Ibáñez I, Olivares R, et al (2001) Control of secretion by mitochondria depends on the size of the local [Ca2+] after chromaffin cell stimulation. Eur J Neurosci 13: 2247–2254. 11454028 10.1046/j.0953-816x.2001.01602.x

[58] Montero M, Alonso MT, Carnicero E, Cuchillo-Ibáñez I, Albillos A, et al (2000) Chromaffin-cell stimulation triggers fast millimolar mitochondrial Ca2+ transients that modulate secretion. Nat Cell Biol 2: 57–61. 10655583 10.1038/35000001

